# Multimodal imaging diagnosis of multiple myeloma with renal involvement: a case report

**DOI:** 10.3389/fonc.2026.1881507

**Published:** 2026-07-14

**Authors:** Lu-Lu Zou, Yuan-Yuan Ma

**Affiliations:** 1 Department of Ultrasound,Yichang Central People’s Hospital (First Clinical Medical College of Three Gorges University), Yichang, Hubei, China; 2 Department of Ultrasound, The First Affiliated Hospital of Shenzhen University Health Science Center, Shenzhen Second People’s Hospital, Shenzhen, China

**Keywords:** case report, multimodal imaging, multiple myeloma, prognosis, renal mass

## Abstract

**Background:**

Extramedullary renal involvement secondary to multiple myeloma (MM) is extremely rare and prone to preoperative misdiagnosis. Its clinical and imaging manifestations have not been systematically summarized, leading to frequent preoperative misdiagnosis. To summarize the multimodal imaging characteristics and clinical diagnostic value of MM with renal involvement through a typical case report, so as to improve the understanding and early recognition rate of this disease.

**Case presentation:**

A 67-year-old male patient with left lumbodorsal pain was examined by multimodal imaging. Ultrasound and contrast-enhanced ultrasound suggested a malignant retroperitoneal mass adjacent to the left kidney. Computed tomography showed extensive invasion of the left renal lesion. Emission computed tomography indicated significantly decreased left renal function. ^18^F-Fluorodeoxyglucose positron emission tomography-computed tomography identified systemic multi-site involvement. Pathological biopsy confirmed anaplastic plasma cell tumor. The patient was finally diagnosed with light-chain MM complicated with renal involvement (Durie-Salmon Stage IIIB, R-ISS Stage III high-risk) and received multi-line chemotherapy, but died of severe infection 5 months after diagnosis.

**Conclusions:**

Multimodal imaging plays an irreplaceable role in the accurate diagnosis, lesion evaluation, and biopsy guidance of MM complicated with renal involvement. It can effectively reduce misdiagnosis and provide important evidence for clinical treatment and prognosis assessment.

## 1 Introduction

Multiple myeloma (MM) is a complex hematological malignancy characterized by the proliferation of clonal plasma cells in the bone marrow, often accompanied by the presence of a monoclonal immunoglobulin protein. The International Myeloma Working Group (IMWG) has established diagnostic criteria that are pivotal in the identification and management of MM. These criteria include bone marrow infiltration by ≥10% clonal plasma cells and the presence of myeloma-defining events, such as CRAB criteria (hypercalcemia, renal failure, anemia, and/or lytic bone lesions) or biomarkers indicating imminent organ damage (1). MM accounts for approximately 10% of all hematological malignancies (2). Extramedullary disease (EMD), characterized by the infiltration of myeloma cells into soft tissues beyond the bone marrow. The reported incidence of EMD varies significantly across studies, likely due to differences in diagnostic methodologies. Among newly diagnosed MM patients, the incidence of EMD is reported to range from 0.5% to 4.8%, whereas in cases of relapsed or refractory MM, the incidence ranges from 3.4% to 14% (3). The detection rate of EMD has gradually increased, correlating with prolonged MM survival and the expanded use of sensitive imaging technologies such as positron emission tomography-computed tomography (PET-CT) (3). The development of EMD may be associated with increased invasiveness of plasma cells, potentially resulting from alterations in the bone marrow microenvironment, impaired homing of plasma cells, and enhanced neovascularization capabilities (4).EMD preferentially involves the head and neck region, particularly the upper respiratory tract (5). In contrast, renal involvement is an extremely rare type of EMD, which poses prominent diagnostic challenges. It lacks specific clinical symptoms and typical imaging manifestations; it is easily misdiagnosed as primary renal carcinoma and other retroperitoneal neoplasms based on conventional radiological findings alone. Combined with the rarity of this disease entity, clinicians often lack sufficient clinical experience for accurate early identification. Once EMD develops in MM patients, it generally predicts limited response to conventional chemotherapy and unfavorable overall prognosis. Various imaging modalities play complementary roles in evaluating EMD: ultrasound (US) and computed tomography (CT) can clarify the size, shape, and invasion range of the mass; ^18^F-fluorodeoxyglucose (^18^F-FDG) PET-CT can detect systemic lesions based on cellular metabolism. Herein, we report a rare case of MM complicated with renal involvement, diagnosed via multimodal imaging with complete documentation of four hospitalization courses. This case aims to summarize the diagnostic features and clinical significance of this entity to improve early recognition.

## 2 Case presentation

### 2.1 Four hospitalization courses and clinical management

#### 2.1.1 First hospitalization (March 2-30, 2025, department of urology → hematology)

A 67-year-old male was admitted to the Department of Urology in March 2025 with a chief complaint of “dull left lumbar pain for over 1 month” (without chills, fever, or urinary symptoms). He reported weight loss and reduced physical strength since symptom onset. Enhanced CT at a local hospital had suggested a left renal space-occupying lesion, prompting further evaluation at our institution.

**Figure 1:**
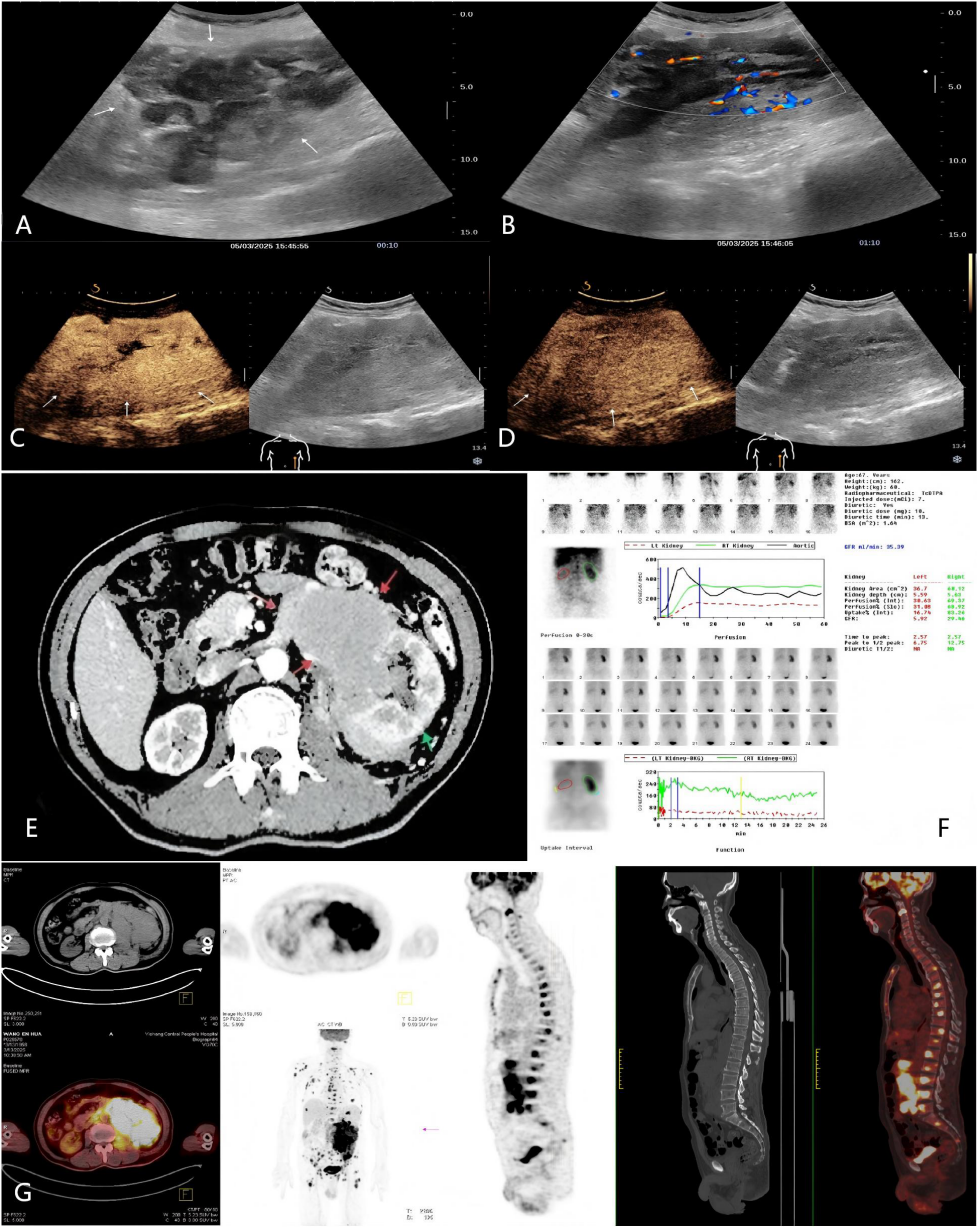
Multimodal imaging of renal extramedullary disease. **(A)** Conventional US showed a heterogeneous hypoechoic mass adjacent to the left kidney without calcification, with irregular shape and unclear boundaries (white arrow indicates the mass boundary); **(B)** Color doppler US showed abundant blood flow signals in the mass; **(C)** CEUS showed the mass exhibited high arterial-phase enhancement: heterogeneous enhancement in early phase. A small area of perfusion defect can be observed in the center of the mass; **(D)** In the late phase, contrast washout initiated earlier within the lesion relative to the renal parenchyma, whereas full clearance of microbubbles was delayed, leading to heterogeneous mild hypoenhancement of the mass; **(E)** CTU in the arterial phase showed that the left renal mass infiltrated the surrounding tissues with obvious exudation (red arrow indicates the mass, and green arrow indicates the left kidney); **(F)** ECT showed significantly reduced blood perfusion of the left kidney (red area), and normal blood perfusion of the right kidney(green area); **(G)** ^18^F-FDG PET-CT fusion image showed abnormal metabolic increase in the left abdominal mass and multiple bones of the whole body, consistent with the manifestations of MM with extramedullary infiltration and bone disease.

On initial workup, conventional US revealed an irregular, ill-defined, heterogeneous solid mass with mixed echogenicity in the retroperitoneum adjacent to the left kidney; color Doppler flow imaging demonstrated abundant blood flow signals within the lesion (**Figures 1A, B**). For contrast-enhanced ultrasound (CEUS) examination, a total of 2.0 mL sulfur hexafluoride microbubble contrast agent was administered via intravenous bolus injection. CEUS demonstrated intense hyperenhancement of the mass in the arterial phase, with an earlier onset of washout yet prolonged full microbubble clearance (**Figures 1C, D**), suggestive of a malignant retroperitoneal solid mass adjacent to the left kidney. Subsequent 64-slice spiral contrast-enhanced abdominal multi-phase computed tomography urography (CTU) was performed. Non-ionic iodinated contrast medium was used for intravenous injection with a standard clinical dose. The scan included non-contrast phase, arterial phase, venous phase and delayed phase. Axial images were reconstructed with a slice thickness of 5.0 mm and an increment of 5.0 mm using the B30f medium-smooth kernel, with a field of view of 337 mm. CTU showed a large irregular left renal mass measuring approximately 8.9 cm × 12.1 cm with blurred perilesional fat planes and exudation, involving the left renal pelvis, ureter, and psoas major muscle. The lesion was supplied by the left renal artery, with segmental invasion of the left renal vein and left iliac vein (**Figure 1E**), consistent with a left renal neoplasm with extensive invasion.

An emission computed tomography (ECT) renal dynamic imaging was performed using 99mTc-DTPA at a dose of 7 mCi to assess renal function: the arterial perfusion phase showed a flat left renal perfusion curve; the functional phase showed faint visualization of the left kidney (glomerular filtration rate [GFR], 5.92 ml/min) and normal right renal function (GFR, 29.46 ml/min); the total GFR of both kidneys was 35.39 ml/min (**Figure 1F**), indicating impaired left renal blood perfusion and function.

**Figure 2:**
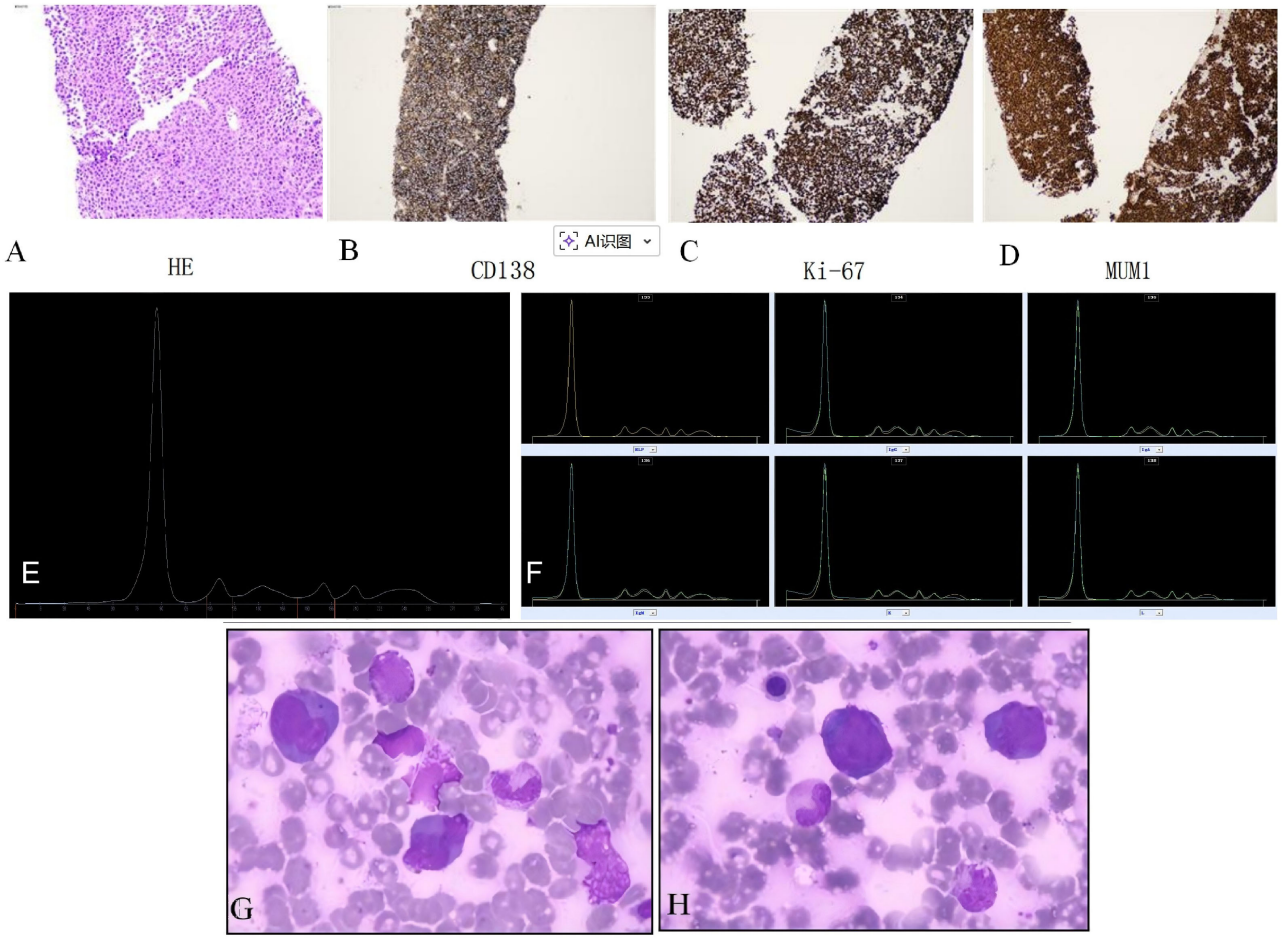
Pathological features, bone marrow cytology and electrophoretic diagnostic evidence of light-chain MM with renal involvement. **(A)** Histopathological and immunohistochemical findings of the renal anaplastic extramedullary plasmacytoma (×40). Hematoxylin and Eosin staining showed dense infiltration of anaplastic plasma cells with irregular nuclei and abundant cytoplasm, arranged adjacent to small blood vessels; **(B)** Immunohistochemical staining for CD138 was positive, confirming the plasma cell origin of the tumor cells; **(C)** Ki-67 staining showed a labeling index of approximately 90%, indicating extremely high proliferative activity of the tumor cells; **(D)** Immunohistochemical staining for MUM1 was strongly positive, further confirming the diagnosis of plasma cell neoplasm. **(E)** Serum protein electrophoresis tracing: The gamma globulin region presents a smooth polyclonal waveform without discrete sharp monoclonal M-protein spike, proving no intact heavy-chain immunoglobulin production; **(F)** Immunofixation electrophoresis: No abnormal clonal bands were identified in IgG, IgA, and IgM heavy chain lanes, consistent with the serum protein electrophoresis result of absent intact M-protein. Combined bone marrow histology, cytogenetic and electrophoretic data establish the diagnosis of light-chain MM according to IMWG criteria; **(G, H)** Bone marrow aspiration smear showed that abnormal tumor cells accounted for 25.0% with binucleated, trinucleated and petaloid nuclei, consistent with the morphological features of MM.

Following these imaging studies, US-guided percutaneous core needle biopsy of the left retroperitoneal mass was performed. Pathological consultation at a higher-level hospital confirmed anaplastic plasma cell tumor, with two concurrent diagnoses: (1) anaplastic MM; (2) anaplastic extramedullary plasmacytoma. Immunohistochemical results were as follows: cluster of differentiation (CD) 138 (+), multiple myeloma oncogene 1 (MUM1) (+), Ki-67 (labeling index approximately 90%), tumor protein 53 (TP53) (strongly positive), pan-cytokeratin (-), S-100 protein (-), and CD20 (-) (**Figures 2A–D**).

To assess systemic disease burden, ^18^F-FDG PET-CT was performed after pathological confirmation. The patient fasted for 6 hours before the examination, and the pre-injection blood glucose level was controlled within the normal range. A total activity of 7.07 mCi ^18^F-FDG was administered intravenously, and whole-body scanning was performed 60 minutes after injection. Iterative reconstruction and attenuation correction were applied for image reconstruction. Imaging showed an abnormal hypermetabolic soft tissue mass in the left abdomen (maximum standardized uptake value [SUVmax], 22.7) involving the left kidney, psoas major muscle, and iliopsoas muscle; multiple hypermetabolic enlarged lymph nodes in the splenic hilum, retroperitoneum, and inguinal region (SUVmax, 19.1); and extensive hypermetabolic bone destruction involving the mandible, humeral heads, ribs, spine, and pelvis (SUVmax, 15.1) (**Figure 1G**). These findings were consistent with MM with renal involvement.

For definitive diagnosis and treatment planning, bone marrow aspiration and biopsy were performed. Myelogram showed 25.0% myeloma cells; flow cytometry identified 10.6% abnormal plasma cells with the phenotype: CD38dim(+), CD138(+), CD19(-), CD56(+), and cytoplasmic kappa (cKappa)(+). Laboratory studies revealed an elevated serum free kappa (κ) light chain (236.93 mg/L) and an elevated κ/λ ratio (11.435); serum protein electrophoresis demonstrated no discrete monoclonal M-protein spike within the gamma globulin zone, and immunofixation electrophoresis detected no clonal heavy-chain bands for IgG, IgA, or IgM (**Figures 2E, F**). Per the IMWG consensus, true non-secretory multiple myeloma is defined by normal serum free light chain levels, so the markedly elevated clonal free κ light chains in this case rule out non-secretory myeloma and confirm light-chain MM. Genetic testing demonstrated TP53 gene mutation, 1q21 amplification (positive rate 65%), and complex karyotype abnormalities (**Figures 2G, H**).

Based on multimodal imaging and pathological findings, the final diagnosis of light-chain MM complicated with renal involvement was confirmed. According to the Durie-Salmon staging system, the patient was classified as Stage IIIB due to heavy tumor burden, extensive bone lesions and myeloma-related renal dysfunction. In accordance with the Revised International Staging System (R-ISS) criteria integrated with serum biomarkers and cytogenetic results, the patient was stratified as R-ISS Stage III (high-risk).The patient was transferred to the Department of Hematology on March 11. Chemotherapy with the bortezomib + lenalidomide + dexamethasone (VRD) regimen was initiated on March 19, combined with symptomatic treatments including uric acid-lowering, hepatoprotective, and nephroprotective therapies. This regimen was administered on a standard 21-day cycle. After one cycle of VRD therapy, the patient’s lumbodorsal pain was partially relieved. Repeated laboratory tests showed persistently elevated serum free kappa light chain, creatinine and inflammatory indicators. No repeated imaging examination was performed after the first cycle of chemotherapy. Combined with the patient’s high-risk cytogenetic profiles, extensive extramedullary lesions and unsatisfactory serological response to standard VRD, we decided to switch to an alternative regimen for subsequent treatment. His abdominal pain was relieved, and he was discharged after completing the first cycle of chemotherapy.

#### 2.1.2 Second hospitalization (April 4-May 28, 2025, department of hematology)

The patient was readmitted two weeks after discharge with fever (maximum temperature 39°C) and diarrhea, accompanied by dry cough and fatigue. Laboratory examinations showed leukopenia (2.42×10^9^/L), aggravated anemia (hemoglobin 97 g/L), elevated serum creatinine (294.2 μmol/L), and elevated C-reactive protein (211.0 mg/L). He was diagnosed with respiratory syncytial virus infection, Aspergillus niger pneumonia, and severe immunodeficiency.

Empirical anti-bacterial, anti-fungal, and anti-viral therapies were administered initially. Given the patient’s severe immunodeficiency and active life-threatening fungal infection, reduced drug dosages were applied to minimize severe myelosuppression. Considering the suboptimal efficacy of the previous VRD regimen, chemotherapy was switched to the dexamethasone + vincristine + doxorubicin (VAD) regimen on April 14. During this course, the respiratory infection was gradually controlled, but anemia and renal dysfunction continued to progress. No repeated imaging was conducted in this hospitalization. Due to limited anti-tumor efficacy and poor treatment tolerance, another regimen adjustment was made for the next cycle. He was discharged in stable condition after completing treatment.

#### 2.1.3 Third hospitalization (June 9-June 30, 2025, department of hematology)

The patient was admitted for scheduled chemotherapy due to the suboptimal response to the prior two regimens. Re-evaluations showed anemia (hemoglobin 72 g/L), hypoalbuminemia (31.75 g/L), hypocalcemia (2.00 mmol/L), and persistent hypoimmunoglobulinemia. Follow-up CT showed a slight reduction in the size of the left renal mass with stable bone lesions.

Chemotherapy with the carfilzomib + pomalidomide + dexamethasone + liposomal doxorubicin regimen was initiated on June 13, supplemented with calcium replacement, albumin infusion, and immune-enhancing therapy. Chemotherapy-induced myelosuppression occurred during hospitalization and was managed with hematopoietic growth factors and blood transfusion. A follow-up non-contrast CT revealed a mild reduction in the size of the left renal mass compared with the baseline measurement (8.9 cm × 12.1 cm). The overall size change was mild and could not be further quantified with precise Response Evaluation Criteria in Solid Tumors (RECIST) diameter values, as only routine plain CT was performed without standardized multi-phase enhanced scanning. All osseous lesions remained stable on CT imaging. Nevertheless, hypoalbuminemia, hypocalcemia and renal impairment persisted. The disease was still refractory to this combined salvage regimen, so we continued to optimize the treatment plan.The patient was discharged in stable condition.

#### 2.1.4 Fourth hospitalization (July 3-August 9, 2025, department of hematology)

The patient was readmitted with recurrent fever, aggravated cough, and poor mental status. The patient received repeated rescue treatments including symptomatic support, anti-infection and combined chemotherapy. Laboratory examinations revealed severe pancytopenia (lymphocyte count 0.13×10^9^/L), elevated serum creatinine (176.7 μmol/L), and elevated C-reactive protein (59.4 mg/L). CT showed increased exudation around the left renal mass and aggravated pulmonary infection.

His condition rapidly deteriorated due to uncontrolled severe infection despite aggressive supportive care. The patient suffered from combined respiratory syncytial virus infection and invasive Aspergillus niger pneumonia. The progressive and refractory infection was the direct cause of his final deterioration. He was discharged with stable vital signs after symptomatic treatment, but unfortunately he passed away shortly after discharge in early August 2025, five months after the initial diagnosis.

Laboratory findings are summarized in **Table 1**, and the dynamic changes of key clinical indicators across the four hospitalizations are presented in **Table 2**.

**Table 1:** Summary of Laboratory Examination Results.

Examination category	Index item	Result/performance	Reference range/clinical significance
Bone marrow examination	Myeloma cell ratio	25.0%	>10% supports MM diagnosis
	Abnormal plasma cell ratio (flow cytometry)	10.6%	Increased, suggesting clonal plasma cell proliferation
	Abnormal plasma cell phenotype	CD38dim+CD138+CD19-CD56+, cKappa monoclonality	Typical MM phenotype
	Bone marrow biopsy	Slightly increased plasma cells, heterogeneous proliferation	Supports MM diagnosis
Serological examination	Free κ light chain	236.93 mg/L↑	3.3~19.4 mg/L
	Free λ light chain	20.72 mg/L	5.7~26.3 mg/L
	κ/λ ratio	11.435↑	0.26~1.65
	Free light chain difference	216.21 mg/L↑	<50 mg/L
	Serum protein electrophoresis + immunofixation electrophoresis	No obvious intact monoclonal M protein band	Consistent with light-chain MM
	β2-microglobulin	8.61 µg/mL↑	1.0~3.0 µg/mL
Renal function	Creatinine (Crea)	Up to 294.2 µmol/L↑	59~104 µmol/L
	Endogenous creatinine clearance rate (Ccr)	As low as 4.56 mL/min↓	>90 mL/min
	24-hour urine protein quantification	0.72 g/24h↑	<0.15 g/24h
	Urine κ/λ ratio	14.236↑	Suggests light chain renal damage
Anemia indicators	Hemoglobin	As low as 43 g/L↓	130~175 g/L (male)
	Red blood cell count, hematocrit	Continuously decreased	Suggests severe anemia
Liver function	Alanine Transaminase	Up to 207.7 U/L↑	<40 U/L
	Aspartate Transaminase	Up to 188.3 U/L↑	<40 U/L
Immune function	IgG/IgA/IgM	All significantly reduced	Normal plasma cell function is inhibited
	Lymphocyte count	As low as 0.13×10^9^/L↓	1.1~3.2×10^9^/L
Pathology and immunohistochemistry	Pathological diagnosis	Anaplastic plasma cell tumor	Consulted by Wuhan Union Hospital
	CD138	+	Plasma cell marker
	MUM1	+	Plasma cell marker
	Ki-67	About 90%	High proliferative activity
	TP53	Strongly positive	Suggests TP53 abnormality
Fish detection	1q21 amplification	Positive rate 65%↑	High-risk prognostic factor
	t(4;14)	Equivocal	High-risk prognostic factor
Gene detection	TP53 gene mutation	Missense mutation (DNA-binding domain)	High-risk prognostic factor
Chromosome karyotype	Complex karyotype abnormalities	add(1)(q21), add(14)(q32), -13, -14, -16, -22, etc.	Suggests high risk

↑ = higher than reference range; ↓ = lower than reference range

**Table 2:** Dynamic Changes of Key Laboratory Indicators During Four Hospitalizations.

Examination index	First hospitalization (mar 2025)	Second hospitalization (apr 2025)	Third hospitalization (june 2025)	Fourth hospitalization (jul 2025)	Reference range
Hemoglobin (g/L)	113↓	97↓	72↓	74↓	130~175 (male)
Platelet (×10^9^/L)	85↓	55↓	177	167	100~300
Leukocyte (×10^9^/L)	1.99↓	2.42↓	4.17	4.24	3.5~9.5
Lymphocyte count (×10^9^/L)	0.49↓	-*	-	0.13↓	1.1~3.2
Creatinine (μmol/L)	168↑	294.2↑	139.2↑	176.7↑	59~104
C-reactive protein (mg/L)	29.9↑	211.0↑	30.3↑	59.4↑	0~8
Albumin (g/L)	35.72↓	33.68↓	31.75↓	24.51↓	40~55
IgG (g/L)	7.12↓	3.77↓	6.16↓	2.79↓	7.0~16.0
β2-microglobulin (μg/mL)	8.61↑	-	-	7.07↑	1.0~3.0

*: “-”indicate that the corresponding laboratory tests were not performed during the corresponding hospitalization period according to the clinical arrangements at that time.

↑ = higher than reference range; ↓ = lower than reference range

## 3 Discussion

MM is a clonal plasma cell malignancy originating from terminally differentiated plasma cells. In most MM patients, plasma cell proliferation is confined to the bone marrow cavity, but some patients may develop extramedullary growth, termed EMD (6). EMD represents an aggressive form of MM characterized by increased tumor independence from the bone marrow microenvironment, leading to extramedullary expansion of plasma cells (7). Accumulating evidence suggests that multiple genetic abnormalities, impaired plasma cell homing, enhanced tumor aggressiveness, and immune evasion synergistically promote extramedullary spread in MM (6).

Renal EMD represents a rare clinical entity with limited published cases. We have summarized published cases of secondary renal disease in MM — consistent with the secondary renal mass presented in our work — in **Table 3**. Collectively, the scarcity of such infiltrating lesions renders them extremely rare in clinical practice.

**Table 3:** A Review of Published Literature on The Renal EMD in MM was Shown.

Cas no.	First author, year	Nation	Age(y)/Sex	EMD involvement	Imaging methods and characteristics	MM type	Therapy	Prognosis
1	Su-Hong Kim et al, 2003 (8)	Korea	F/44	Right kidney	CT: Right kidney mass	IgG-lambda	Radical resection of the right kidney + Hyper-CVAD chemotherapy + Local radiotherapy	Survival without disease for 3 months after recurrence
2	Köse M et al, 2015 (9)	Turkey	M/53	Left upper pole of the kidney + Head of the pancreas + Left adrenal gland + Multiple skin lesions + Lungs + Liver + Spleen + Lymph nodes + Bones	CT: Homogeneous solid mass in the pancreatic head + Left adrenal gland + Left renal upper pole mass + Enlarged liver and spleen	IgG kappa	VAD×1 → CHOP×2	The mass has significantly shrunk and treatment is continuing.
3	Zhang MJ et al, 2015 (2)	China	M/76	Intracranial extension and bilateral renal infiltration	MRI(head):an osteolytic skull lesion with intracranial extension CT(abdomen):a large tumor mass extending around and into the kidneys	IgG (39.25 g/L) + Bence Jones protein (+), bone marrow plasma cells 4%	CTD×2	It almost completely disappeared after 4 months.
4	Ilyas U et al, 2022 (10)	America	M/56	Retroperitoneal (pararenal)	POCUS: IVC (inferior vena cava) continues to collapse; MRI: A retroperitoneal mass is compressing the IVC.	IgG lambda	Vasopressin + High-dose Dex + CTX → CyBorD	Discontinue vasopressin, and be discharged for continued chemotherapy.
5	Zafar Sheikh A et al, 2023 (11)	Pakistan	M/62	Right kidney	CT: Right kidney enlargement (suspected RCC); MRI: Homogeneous signal + restricted diffusion + renal vessels/IVC encirclement (suspected lymphoma)	Kappa-restricted MM	CTD×2	Febrile neutropenia + DIC + AKI → Death
6	Kavgaci G et al, 2024 (12)	Turkey	M/64	Right lower pole of the kidney + renal hilum + bilateral testicles + pelvic recurrence	CT:ight lower pole of the kidney + renal hilum mass	IgG kappa	VDT-PACE → Auto-SCT → Len+Ixa	15+ months of sustained remission
7	Tsanava K et al, 2026 (13)	Georgia	F/65	Right kidney+liver	CT:Right kidney mass+ Multiple lesions in the liver	NR	chemotherapy and hemodialysis	After a 2-month follow-up, the liver and kidney masses showed significant reduction.
8	Jamet B et al, 2025 (14)	France	F/63	Both kidneys	^18^F-FDG PET-CT	IgG type	NR	NR

NR, Not Reported; MM, Multiple Myeloma; EMD, Extramedullary Disease; AKI, Acute Kidney Injury; RCC, Renal Cell Carcinoma; DIC, Disseminated Intravascular Coagulation; POCUS, Point-of-care Ultrasound; IVC, Inferior Vena Cava; Hyper-CVAD, Hyperfractionated Cyclophosphamide, Vincristine, Adriamycin, Dexamethasone; CHOP, Cyclophosphamide, Doxorubicin, Vincristine, Prednisone; CTD, Cyclophosphamide, Thalidomide, and Dexamethasone; Dex, Dexamethasone; CTX, Cyclophosphamide; CyBorD, Cyclophosphamide, Bortezomib, and Dexamethasone; Auto-SCT, Autologous Stem Cell Transplantation; VDT-PACE, Bortezomib, Dexamethasone, Thalidomide, Cisplatin, Doxorubicin, Cyclophosphamide, and Etoposide; Len, Lenalidomide; Ixa, Ixazomib. → indicates changes in treatment regimens and disease course

This case is a rare light-chain MM with renal involvement, with complete records of four hospitalizations, which fully reflects the aggressive characteristics of the disease. In the diagnosis stage of this case, in addition to the routine use of CT and PET-CT, innovative diagnostic modalities including CEUS were also adopted, and US-guided biopsy was subsequently performed. CEUS is a method that enhances the blood flow harmonic signals in the human body by injecting ultrasound contrast agent (microbubbles) through the vein, and it enables real-time dynamic observation of the microvascular perfusion information of the target tissue. Therefore, compared with previous reports, the imaging methods used in this case are comprehensive and novel, especially in the ultrasound examination aspect.This patient had abdominal pain and US revealed a space-occupying lesion near the left kidney in the retroperitoneal area. The lesion was an irregular, mixed echo mass with varied internal echogenicity and abundant blood flow, matching ultrasonographic characteristics of EMD. Trenker et al.’s analysis of 24 EMD lesions showed all were hypoechoic on B-mode US, with 66.6% having smooth margins and 33.3% irregular margins (15). US further revealed marked arterial hyperenhancement of the lesion. Washout began earlier than in the adjacent renal parenchyma, yet full microbubble clearance was prolonged in the late phase, resulting in heterogeneous mild hypoenhancement. This distinct perfusion feature was previously documented in 83.3% of myelomatous extramedullary lesions by Trenker et al. and Chen et al., and is attributed to abundant disorganized intralesional angiogenesis (15, 16).US is invaluable for EMD diagnosis due to its non-invasive, real-time, high-resolution imaging, which allows quick identification and localization of abdominal masses (16). CEUS highlights necrotic and viable tissue areas, aiding accurate biopsy guidance and avoiding the pitfalls of blind puncture. Additionally, US-guided biopsy ensures precise needle placement into viable tissue, safeguarding surrounding blood vessels (17). This approach allows obtaining high-quality pathological specimens with minimal risk, providing reliable evidence for subsequent diagnostic classification and targeted therapy. As highlighted in the aforementioned literature, US plays a critical role in initial screening during diagnosis, guiding further diagnostic modalities such as CT, nuclear magnetic resonance (MRI) or biopsy, serving as an indispensable “gatekeeper” in the diagnostic process for abdominal masses.

However, US has limitations: its scanning field is limited, making it difficult to evaluate systemic lesions such as MM. In this case, US could only focus on the local mass, renal involvement as an initial manifestation of MM is not taken into account and the possibility of MM was not considered initially due to the rarity of renal involvement.

Following an initial diagnosis of renal carcinoma, a CT scan showed a large, irregular mass in the left kidney affecting the renal pelvis, ureter, and psoas muscle. The mass was supplied by the left renal artery and involved segments of the left renal and iliac veins. ECT confirmed reduced blood flow in the left kidney, aligning with the vascular involvement seen on CT. CT enhanced scanning excels in confirming the blood supply source of an extramedullary lesion and revealing its invasive behavior, while also explaining the functional perfusion reduction seen in ECT by showing renal pelvis and ureteral involvement. Its detailed imaging accurately localizes complex lesions involving blood vessels, collecting systems, and muscles, offering crucial evidence for clinical risk assessment, targeted therapy decisions, and radiotherapy planning.

Notably, CT has limitations in evaluating bone marrow lesions, as it mainly shows the impact of myeloma on trabecular and cortical bone, not the marrow lesions. In long bones, CT might detect abnormalities if myeloma cells fill the marrow cavity, but evaluating MM infiltration in trabecular-rich areas like vertebrae is difficult due to the bone structure and possible degenerative changes or osteoporosis (7). In contrast, ^18^F-FDG PET-CT, as a safe and non-invasive imaging modality, can reflect changes in glucose metabolism prior to bone destruction and directly demonstrate lesion activity. IMWG standards have been updated, indicating that the presence of>1 osteolytic lesion assessed by CT, whole-body low-dose CT, or PET-CT, along with>1 focal bone marrow lesion (≥5 mm) on MRI, is sufficient to confirm osteopathy (18, 19).

In addition, PET-CT is an effective systemic tool for detecting extramedullary lesions, crucial in diagnosing this case. It showed heightened metabolic activity in various bones and lymph nodes, along with a soft tissue mass in the left abdomen affecting nearby structures, indicating MM with extramedullary infiltration and widespread bone invasion. ^18^F-FDG PET-CT is highly valuable for diagnosing MM, as it effectively maps systemic bone marrow lesions and detects extramedullary infiltrative lesions. As a combination of functional imaging and anatomical imaging, PET-CT enables whole-body imaging in a single session, distinguishes metabolic active sites from non-active sites of diseases, provides earlier efficacy evaluation, and can predict prognosis (20). PET-CT measures FDG uptake, thereby quantifying disease burden changes over time (20, 21). Recent studies by Zanwar et al. demonstrated that PET-based metabolic response assessment holds significant prognostic value in EMD patients (22). In this case, all lesions presented remarkably high ^18^F-FDG uptake (SUVmax: renal mass = 22.7, lymph nodes = 19.1, bone lesions = 15.1). In the diagnosis of multiple myeloma, PET-CT exhibits higher SUVmax values, particularly prominent in detecting EMD. Studies indicate that EMD typically demonstrates high metabolic activity on PET-CT scans, with an average SUVmax of 8.4. This hypermetabolism is closely associated with disease aggressiveness and poor prognosis (23). Furthermore, in the distribution patterns of extramedullary multiple myeloma, hematogenous/lymphatic dissemination is more common, whereas direct bone marrow extension is less frequent. These distinct distribution patterns are also reflected in differences in SUVmax values, with lesions exhibiting direct bone marrow extension demonstrating significantly higher SUVmax values than those with hematogenous/lymphatic dissemination (23). Combined with the high Ki-67 index and high-risk cytogenetic abnormalities in this patient, the extremely high metabolic activity fully accounts for the aggressive disease course.

The imaging differential diagnosis for this case mainly includes renal cell carcinoma (RCC), renal lymphoma and retroperitoneal sarcoma. The findings from ultrasound and CEUS (rapid approach and rapid washout with prolonged complete clearance, high enhancement) as well as the local findings from CTU (venous invasion, renal artery supply, “rapid approach and rapid retreat” richly vascularized enhancement, surrounding exudation) closely resemble RCC, leading to the initial diagnosis of renal cancer and subsequent biopsy. Renal EMD on traditional CT/MRI often cannot reliably distinguish from RCC (13, 24). The comprehensive assessment of FDG PET-CT revealed key distinguishing clues: ① The widespread symmetrical multilevel osteolytic destruction pattern points to MM rather than RCC metastasis (25); ② Multiple station lymph node involvement points to lymphoid proliferation/lymphoplasmacytic tumor rather than sarcoma (sarcoma rarely metastasizes via the lymphatic pathway and lacks fat components to rule out liposarcoma) (26). It is worth noting that intraluminal vascular invasion is a key feature for differentiating lymphoma. The literature clearly states that true intraluminal invasion in lymphoma is extremely rare (27). However, this disease has no clear specificity in imaging, and diagnosis still relies on biopsy and multidisciplinary collaboration (28). This case exemplifies the complementary utility of multimodal imaging in diagnosing MM with EMD. US facilitates the initial localization of lesions and aids in guiding biopsy procedures. CT provides detailed anatomical delineation of lesions, their spatial relationships with adjacent structures, and the extent of invasion. ECT offers quantitative assessment of renal function, while PET-CT evaluates the systemic distribution and metabolic activity of lesions. Each imaging modality presents unique advantages, collectively contributing to a comprehensive understanding of the disease.

The patient was staged as Durie-Salmon Stage IIIB and R-ISS Stage III (high-risk). He exhibited several additional high-risk factors, including a TP53 mutation, 1q21 amplification, and a Ki-67 index of 90%, leading to rapid disease progression. These findings collectively indicate a highly aggressive disease course. Literature reports suggest that EMD patients with high-risk cytogenetic abnormalities have poorer prognosis and exhibit poor response to conventional chemotherapy (29). Standard VRD is a widely used first-line regimen for newly diagnosed multiple myeloma. However, this conventional triplet regimen shows limited efficacy in high-risk patients with EMD, especially those complicated with adverse cytogenetic abnormalities such as TP53 mutation and 1q21 amplification.For this high-risk case with renal EMD and progressive disease under VRD treatment, individualized therapeutic strategies should be considered. Early switch to second- or third-generation proteasome inhibitors combined with immunomodulatory drugs or monoclonal antibodies is recommended to improve anti-tumor activity against extramedullary lesions. For patients complicated with recurrent and severe infections, reduced-dose chemotherapy combined with long-term anti-infection prophylaxis and hematopoietic support therapy is necessary to balance anti-tumor effect and treatment safety.Autologous hematopoietic stem cell transplantation was not adopted in this patient due to advanced age, persistent renal dysfunction and repeated life-threatening infections, which would lead to excessively high transplantation-related mortality. For similar refractory high-risk myeloma with soft tissue EMD, sequential salvage regimens and combined targeted agents are the main alternative treatment options in clinical practice.

The initial VRD regimen demonstrated limited efficacy, highlighting the refractory characteristics of high-risk EMD. The patient had repeated severe infections during four hospitalizations, which is the main cause of death. The root cause is severe immune deficiency caused by the disease itself and chemotherapy. Clinicians should prioritize infection prevention and promptly initiate anti-infective therapy in similar high-risk EMD patients. Patients with advanced MM universally suffer from severe immune dysfunction. Malignant clonal plasma cells suppress normal bone marrow hematopoietic function and impair humoral and cellular immunity, leading to persistent hypogammaglobulinemia and decreased lymphocyte counts. This profound immunodeficiency greatly increases susceptibility to bacterial, viral and fungal infections. Combined with the myelosuppressive effects of multi-line chemotherapy, the risk of refractory and life-threatening infections rises sharply, which is a well-recognized leading cause of death in advanced and high-risk multiple myeloma patients. In this case, the patient developed combined respiratory syncytial virus and invasive Aspergillus niger pneumonia, and eventually died of uncontrolled severe infection, which is consistent with the typical disease progression of advanced MM with severe immune impairment.

## 4 Limitations

This study has several inherent limitations that need to be acknowledged. First, complete surgical pathological specimens were not obtained. The diagnosis was established based on percutaneous core needle biopsy samples rather than surgical resection specimens. Although the biopsy results were sufficient for a definitive diagnosis, limited tissue volume restricted further comprehensive pathological analysis. Second, serial follow-up PET-CT examinations were not available after each cycle of chemotherapy. As a retrospective study conducted according to real-world clinical practice, only routine plain CT scans were arranged for re-evaluation. Therefore, we could not perform quantitative therapeutic response assessment following standard PET-CT-based metabolic criteria and RECIST guidelines. Third, this is a single retrospective case report of rare renal EMD complicated with high-risk MM. The findings have limited generalizability to broader patient populations, and large-sample studies are still needed to verify our conclusions.Fourth, molecular characterization of the tumor remains incomplete. We only detected partial cytogenetic abnormalities including TP53 mutation and 1q21 amplification, while more comprehensive molecular profiling was not conducted due to clinical and technical constraints.

In addition, as a single-center retrospective analysis based on real clinical records, the treatment regimens were formulated according to on-site clinical judgment for this critically ill patient, which may not be fully consistent with standardized protocols from specialized hematology centers.

## 5 Conclusions

This case reports a rare light-chain MM with renal involvement, with complete records of multimodal imaging diagnosis and four hospitalization courses. Multimodal imaging (US→CEUS→CTU→ECT→^18^F-FDG PET-CT) constructs a complete diagnostic chain, which is the key to avoiding misdiagnosis. The four hospitalization courses and dynamic indicator changes further highlight the aggressive characteristics of high-risk EMD. For patients with atypical retroperitoneal space-occupying lesions, clinicians should be alert to the possibility of MM with EMD, and timely adopt multimodal imaging and pathological biopsy to clarify the diagnosis, so as to formulate individualized treatment plans and improve patient prognosis.

## Data Availability

The original contributions presented in the study are included in the article/**Supplementary Material**. Further inquiries can be directed to the corresponding author.
